# Can Machines Find the Bilingual Advantage? Machine Learning Algorithms Find No Evidence to Differentiate Between Lifelong Bilingual and Monolingual Cognitive Profiles

**DOI:** 10.3389/fnhum.2021.621772

**Published:** 2021-03-22

**Authors:** Samuel Kyle Jones, Jodie Davies-Thompson, Jeremy Tree

**Affiliations:** Department of Psychology, Swansea University, Swansea, United Kingdom

**Keywords:** machine learning, bilingualism, cognition, dementia, cognitive decline, executive function, language

## Abstract

Bilingualism has been identified as a potential cognitive factor linked to delayed onset of dementia as well as boosting executive functions in healthy individuals. However, more recently, this claim has been called into question following several failed replications. It remains unclear whether these contradictory findings reflect how bilingualism is defined between studies, or methodological limitations when measuring the bilingual effect. One key issue is that despite the claims that bilingualism yields general protection to cognitive processes (i.e., the cognitive reserve hypothesis), studies reporting putative bilingual differences are often focused on domain specific experimental paradigms. This study chose a broader approach, by considering the consequences of bilingualism on a wide range of cognitive functions within individuals. We utilised 19 measures of different cognitive functions commonly associated with bilingual effects, to form a “cognitive profile” for 215 non-clinical participants. We recruited Welsh speakers, who as a group of bilinguals were highly homogeneous, as means of isolating the bilingualism criterion. We sought to determine if such analyses would independently classify bilingual/monolingual participant groups based on emergent patterns driven by collected cognitive profiles, such that population differences would emerge. Multiple predictive models were trained to independently recognise the cognitive profiles of bilinguals, older adults (60-90 years of age) and higher education attainment. Despite managing to successfully classify cognitive profiles based on age and education, the model failed to differentiate between bilingual and monolingual cognitive ability at a rate greater than that of chance. Repeated modelling using alternative definitions of bilingualism, and just the older adults, yielded similar results. In all cases then, using our “bottom–up” analytical approach, there was no evidence that bilingualism as a variable indicated differential cognitive performance – as a consequence, we conclude that bilinguals are not cognitively different from their monolingual counterparts, even in older demographics. We suggest that studies that have reported a bilingual advantage (typically recruiting immigrant populations) could well have confounded other key variables that may be driving reported advantages. We recommend that future research refine the machine learning methods used in this study to further investigate the complex relationship between bilingualism and cognition.

## Introduction

Dementia is characterised by a continuous and largely irreversible decline in cognitive processing. Patients are diagnosed through showing deficits in cognitive domains such as memory, attention, problem solving, recognition, and language processing (World Health Organization, 2012). While Alzheimer’s disease is the leading cause of dementia, it has also been linked to other pathologies, trauma, and more generally associated with cognitive decline as result of ageing. With an aging population across much of the developed world it follows that the prevalence of dementia will increase and so too the individual and societal costs (Hebert et al., 2001). Due to its multifaceted causes and covariates there is currently no treatment or cure (World Health Organization, 2019), therefore, research into the field has also undertaken a more preventative approach, centred around the concept of cognitive reserve. Cognitive reserve refers to the phenomenon of adaptively allocating cognitive resources to complete tasks more efficiently or to compensate for neural damage and thus mitigate the symptoms of cognitive decline (Kowoll et al., 2016). Greater cognitive reserve has been observed repeatedly to manifest in delayed onset of dementia despite similar levels of pathology (Stern, 2012). What makes cognitive reserve particularly appealing is that it can be modulated through certain cognitively stimulating behavioural activities (Scarmeas and Stern, 2004). While controversial (see Zahodne et al., 2014) bilingualism has been argued to be one such activity that contributes to increased cognitive reserve as well as enhancing cognition among non-clinical individuals (Grant et al., 2014). This bilingual advantage has been demonstrated over multiple cognitive domains and across several age groups with the greatest differences among children and older adults (Bialystok, 2017). However, this view has been contested by more recent empirical studies, calling into question the relevance of bilingualism as a factor of cognitive reserve (Paap et al., 2016). Critics cite the inconsistent findings, failed replications and conflating of the effects of bilingual covariates (immigration status, proficiency in languages, socio-economic status) with the effects of bilingualism itself (Paap et al., 2015b). Alternatively, proponents of the bilingual effects on cognition argue that the failed replications to not take into account the complex nature of bilingualism and use narrow domain specific tasks that are not sensitive enough, on their own, to detect the more salient domain general effect of bilingualism on cognition (Bialystok, 2016).

Our approach was to evaluate the effects of bilingualism and cognitive performance by adopting four key methods: (1) Homogeneous sampling of both monolingual and bilingual groups, with an effort to isolate the variable of bilingualism, (2) Utilising a more holistic set of measures of “bilingual identifiers” to more completely recognise potential variability on issues such as: proficiency, frequency of use and exposure, (3) Utilisation of a within participants cognitive ‘profiling’ approach that entailed testing that spans a number of domain general/domain specific assessment paradigms, (4) Utilisation of machine learning driven analytical techniques, this “bottom-up” approach sought to distinguish bilinguals from monolinguals based on their cognitive abilities across multiple domains. The advantage of the Random Forest machine learning classification algorithm, employed here, over more traditional statistical inference is that it is able to consider the participants’ cognitive performance across multiple tasks simultaneously. By training the algorithm on a wide array of cognitive abilities derived from the same participants it is able to determine whether there is a general cognitive pattern that identifies them as members of a distinct group, while also identifying which domain specific abilities contribute the most to this distinction. While machine-learning algorithms are building momentum in fields such as economics, and medicine, there is limited application in psychology (Dwyer et al., 2018). Therefore, the validity of machine learning techniques as a method of studying complex, multi-dimensional problems in empirical research was also further evaluated.

Bilingualism has been asserted to be an experience capable of changing brain structure and function across multiple cognitive domains, resulting in a shift in how bilinguals recruit cognitive resources that is fundamentally different to monolinguals (Price et al., 1999; Fabbro et al., 2000; Hernandez et al., 2001; Rodriguez-Fornells et al., 2006; Kroll and Bialystok, 2013). This has been shown to manifest in advantages to executive functioning, disadvantages to language processing (Bialystok, 2009), and a delay in the onset of dementia and cognitive decline (see Van den Noort et al., 2019). Retrospective studies have shown bilingual Alzheimer’s Disease (AD) patients can develop symptoms at least up to 5 years later than monolingual patients (Craik et al., 2010; Woumans et al., 2015).

Bilinguals have been also found to outperform monolinguals in cognitive tasks relating to a range of executive functions, including inhibition (Bialystok et al., 2004), task switching (Costa et al., 2008; Green and Abutalebi, 2013), attentional control and working memory (Miyake et al., 2000; Alladi et al., 2013). These advantages have been demonstrated in healthy children (Bialystok, 2011), young adults (Costa et al., 2008), and older adults (Bialystok et al., 2004). This has led to the suggestion that lifelong bilinguals draw on domain-general executive function processes to maintain two simultaneously activated languages (Green, 1998; Barac and Bialystok, 2012). To avoid interference, bilinguals must inhibit one language while engaging the other, resulting in substantial cognitive stimulation during daily communication involving several neural structures (Bialystok, 2016), particularly when switching between languages (Luk et al., 2011). The saliency of the bilingual advantage has been argued to increase with the number of languages spoken (Chertkow et al., 2010) and the proficiency in those languages (Christoffels et al., 2006; Yudes et al., 2011). However, constantly managing multiple competing languages has disadvantages to language processing domains (Bialystok et al., 2009). For example, in tasks assessing vocabulary, language production, and fluency in a single language, bilinguals are found to perform worse than their monolingual counter-parts (Bialystok, 2009). This may be due to the increased time and cognitive resources needed to correctly interpret the context, select the appropriate language, and inhibit the other language before responding (Mägiste, 1979; Ransdell and Fischler, 1987; Katz et al., 2012), or may alternatively be due to reduced relative vocabulary and practise in each language (Michael and Gollan, 2005; Sandoval et al., 2010).

Cognitive reserve (CR) is the process by which the brain adapts and mitigates deterioration of cognitive processes either due to age or disease (Stern, 2009). With such a process in mind, it has been argued that given bilinguals’ experience of continuous cognitive stimulation, this life-skill is considered to be a contributor to increasing cognitive reserve in the same vein as education, and engagement in stimulating social interactions. It therefore follows that if bilingual experience contributes to a boost in an individual’s cognitive reserve, then it would be expected that symptoms of cognitive decline will present later in life. Importantly, bilingualism can be present across a wide spectrum of socio-economic status, educational achievement, and demographics while also potentially sharing identical environments, lifestyles and opportunities as their monolingual peers (Craik et al., 2010). This is especially so in particular cultures where two languages are integrated within the culture of the society (e.g., Wales) and this is a key motivation for the work undertaken here.

Clearly, it is important with such populations to identify the exact parameters under which bilingualism effects cognition and determine the extent to which behavioural factors play a role in the manifestation of cognitive decline, both as a result of pathology and aging.

While bilingualism has a great deal of potential as a mitigator of cognitive decline, it also provides substantial challenges to researchers (Gold, 2016). An examination of the previous literature shows a wealth of contradictory results (Bak et al., 2014; Kousaie et al., 2014; Paap et al., 2015b), with up to 80% of studies investigating the bilingual advantage finding no difference between monolinguals and bilinguals (Paap et al., 2015b). Confounding variables and methodological weaknesses have been implicated as the cause of conflicting findings (Calvo et al., 2016). Specifically, inconsistencies with how bilingualism was defined between studies, with bilinguals being defined through self-identification, frequency of use, exposure, proficiency (Gollan et al., 2011), and age of second language (L2) acquisition.

Studies, including those which find a bilingual effect (Bialystok et al., 2006) as well as those which don’t (von Bastian et al., 2016), often include heterogenous bilingual samples, where the bilinguals in that sample do not consistently speak the same language pairs (Calvo et al., 2016). Despite this being a common practice, very little research has been conducted on its potential confounding effects, and the findings currently available are inconsistent (Barac and Bialystok, 2012; Coderre and van Heuven, 2014; Oschwald et al., 2018; Paap et al., 2015a). Furthermore, critics of the bilingual effect argue that the reliability of prominent and seminal papers is low because of a publication bias favouring small, underpowered studies with statistically significant results, over studies with robust designs and larger samples but which find negative or null effects of a bilingual advantage. The implication being that when larger scale and more comprehensive replications are attempted by different teams, the bilingual effects are rarely reproduced (de Bruin et al., 2015; Paap et al., 2015b, 2016).

As has been discussed earlier, it may well be that early reports of positive effects of bilingualism may in fact reflect other confounds that are driving cross sample performance differences. For instance, bilingual immigrants are regularly compared to the native born monolinguals, this has led to the suggestion that the positive/negative effects of bilingualism may actually be a characteristic of immigrant status. Calvo et al. (2016) detail several traits associated with immigrant status and improved cognition/cognitive reserve that are rarely controlled for in bilingualism research. They argue that depending on the country of origin the immigrant populations from which the bilingual samples are drawn may have a higher or lower socio-economic status than the general, monolingual, native population. Additionally, cultural differences in terms of attitudes to health and community care may affect the point at which individuals seek medical attention, which may impact on the validity of retrospective and prospective studies. Aspects of their language and social experiences may also be unique to immigrant bilingual populations, that would then not apply to bilingualism more generally. Of particular note is age of acquisition of their second language, the context and speed at which a language is learnt, and the exposure to a new culture – all of which have been shown to play a role in modulating cognition and cognitive decline (Bak, 2016). Again, to foreshadow the current work, issues such as these were seen as a major benefit of testing bilinguals in the Welsh context.

Proponents of the bilingual effect have argued that null reports arise not because of the sampling confounds described above, but rather because of a failure to measure cognitive changes in a sufficiently broad fashion. Among others (Kroll and Bialystok, 2013) have written that bilingual effects, when measured by single domain specific tasks, can easily be obfuscated and rendered non-significant by noise. This overly specific focus masks potential cognitive differences occurring on a general level (Friedman and Miyake, 2017; Kroll and Bialystok, 2013; Miyake et al., 2000). This argument emphasises the multi-faceted effects of bilingualism on the brain, strengthening a network of cognitive domains making the system as a whole more efficient and resistant to decline (Bialystok et al., 2009). In this regard, bilingualism is claimed to be similar to other contributing factors of cognitive reserve (e.g., education, social activity) for which it is claimed higher cognitive reserve provides more domain general increases in cognitive resources for the completion of complex tasks. This is generally supported by neuroimaging literature, with bilinguals demonstrating lower brain activation than monolinguals in simple tasks, evidencing efficiency, and also activation of these areas, evidencing compensation. However, it is unclear whether these neurological changes translate into measurable behavioural changes (Paap et al., 2015b), although it does identify a rationale for a more holistic, non-domain specific, approach to accurately explore the extent to which bilinguals differ from their monolingual counter-parts (Hilchey and Klein, 2011)– and this was a key driver for the current study that attempted to understand cognitive changes in our samples from the perspective of a more holistic cognitive ‘profile.’

The current debate centres around two key hypotheses, A) Bilinguals and monolinguals are functionally different in the allocation of cognitive resources in a way that is measurable by cognitive-behavioural tests, and B) Bilingualism mitigates cognitive decline analogous to contributing factors of cognitive reserve. To investigate these two questions, several issues must be addressed, fitting broadly in two categories. (1) as sufficiently robust definition of bilingualism, and (2) selecting measures that reflect the multifaceted effects of bilingualism such that potential domain specific and domain general consequences are recognised.

In service of this first challenge, we have already highlighted that previous studies have often classified bilingual cohorts in such a manner that factors such as immigrant and socio-economic status can introduce numerous confounding variables that may either introduce noise or be implicated as driving the observed effect. Therefore, this study aimed to maximise sample homogeneity by focusing on bilinguals as native, lifelong speakers of one consistent language pair, who were matched across socio-demographic characteristics with their monolingual counterparts – Welsh speakers provided the ideal opportunity to do so. Language factors that are more relative to an individual’s experience (e.g., frequency of use, exposure, proficiency), which nonetheless impact the bilingual advantage, were recorded to provide a more nuanced and conservative proxy for bilingualism. Given the risk of small effect sizes of bilingualism, using a classification that incorporates widely used indicators of bilingualism was hypothesised to help highlight the cognitive effects.

Although these points go some way in appropriately balancing bilingual and mono-lingual populations for comparison, we must also consider issue 2 above. To address this, we undertook testing across a broad range of cognitive measures. In addition, our analyses utilised a novel approach that incorporated machine learning algorithms to differentiate bilinguals from monolinguals based on their cognitive abilities. By implementing machine learning algorithms it is possible to simultaneously analyse cognitive performance across several domains and determine whether there is a recognisable, consistent difference between bilinguals and monolinguals (von Bastian et al., 2016). This “bottom-up approach” effectively reverses the traditional design of bilingualism studies research that prioritises domain specific findings, whereby bilingualism is assessed whether to have a significant impact on a specific cognitive ability or task. Instead, a combination of cluster and classification algorithms is able to take a more holistic view of the data, develop predictive models and identify the most important features of the model.

Recently, interest in the involvement of computational analysis in the fields of psychology and psychiatry is becoming more prevalent as a pragmatic addition to pure significance testing (Paulus et al., 2016). While currently uncommon, machine learning algorithms have also begun to be incorporated into dementia and bilingualism research due to their advantages over more traditional significance tests as a method of analysis for highly dimensional problems (Maroco et al., 2011; von Bastian et al., 2016). Therefore, this study aims to both contribute to the growing body of research assessing the validity of machine learning models in cognitive psychology, as well as evaluate the hypotheses outlined above.

## Methods

### Participants

Data was compiled from 215 non-clinical participants. All participants were screened prior to taking part in the study. If they reported any clinical diagnosis of cognitive impairment or cognitive decline, current or historic, then they were excluded from the study. Ages ranged from 18 to 88 years (*M* = 48.58, *SD* = 22.04). Bilinguals (*N* = 106) spoke Welsh and English and learnt their L2 before adulthood. Monolinguals (*N* = 109) spoke just English and no other language. All participants were recruited in Wales as part of a larger study into cognitive reserve from a database of psychology students studying at Swansea University and members of the general public. Both language groups were matched in English proficiency, education and age, but were significantly different in indicators of bilingualism (Welsh proficiency, frequency of use, and exposure). Student participants received course credits for their time, while members of the public were compensated for their travel costs.

This language group pairing was a particularly attractive demographic to study for a number of reasons. Being a country within Britain, with both Welsh and English languages maintaining official status, Wales is in an opportune position for comparing between bilingual and English-speaking monolingual groups. Wales has the benefit of Government backed infrastructure providing the potential for regular high-quality education and services in both English and Welsh, and has the largest native bilingual demographic, nearly 562,000 (19%) speakers, 318,800 (11%) fluent. It provides a large, and diverse sample across all socio-demographic factors. Additionally, unlike much of the current bilingual research that exists, Welsh bilinguals and monolinguals are identical across every parameter other than language group, with both groups occupying the same environment, educational opportunities, career opportunities and socio-economic status (Clare et al., 2016).

The relationship between Welsh and English languages is a novel one in bilingual research, unlike more common pairs such as, French-English (Kousaie et al., 2014), Chinese-English (Abutalebi et al., 2015), or Japanese-English (Crane et al., 2010), Welsh exists as a minority language under the social dominance of English. While this may then appear to be more similar to the relationships between languages like Basque-French/Basque -Spanish (Lallier et al., 2016), or Catalan- Spanish (Sebastián-Gallés et al., 2012), Welsh and English originate from entirely different linguistic families, Celtic and Germanic, respectively. Therefore, despite sharing an orthographically similar alphabet corresponding to relatively consistent phonemes due to Latin influences, Welsh has a very different grammar structure, as well as the existence of sounds and vocalisation patterns that don’t appear in English.

With that being said, the breadth of lexical information available for Welsh words is not as well developed as in English, hindering inferences related to orthographic similarity, and access to Welsh language testing materials. However, due to the shared history, culture and geographical proximity, it is not unreasonable to assume that lexical attributes such as AoAv, familiarity, even frequency is far more equitable than that of other languages. Welsh-English bilinguals are observed to regularly engage in code-switching with other bilinguals, demonstrating a natural fluency between the two languages (Davies and Deuchar, 2010). Interestingly this has resulted in the development of a high number of loan words that are used in common parlance, making the study of cognate effects particularly viable.

There is some precedence in the use of Welsh-English bilinguals in CR related research. From the previous literature 3 experiments have recently found significant findings of bilingual enhancement among normal samples and samples of Parkinson’s and AD patients in Wales (Khachaturian et al., 2006; Hindle et al., 2015; Clare et al., 2016).

### Materials

Participants first completed a comprehensive computer based questionnaire detailing their demographic, and language background. Participants were assessed using a language background questionnaire which was closely modelled on the Language Experience and Proficiency Questionnaire (LEAP-Q, Marian et al., 2007). This questionnaire measured their subjective proficiency in English and Welsh, age of acquisition, preferred language, level of exposure (e.g., media, socialising, education), frequency of use, and if they had any experience with other languages. Frequency of use, and level of exposure was measured in relation to Welsh, as it is the minority language in a predominantly English speaking culture. It was therefore assumed that all participants regularly engaged and used English as part of their daily routine, and all tasks were completed in English.

Following the questionnaires, participants completed a predominantly computer-based cognitive performance battery. Participants were tested in one 2 hour session, under laboratory conditions. Participants were welcome to breaks of up to 10min between tasks. All participants followed the standard procedures of the individual tests, and the order of the tasks was randomised for each participant. The data consisted of measures from 10 tasks encompassing areas related to language, attention, inhibition, executive control and memory that have previously shown significant bilingual effects (either advantage or disadvantage). These included a lexical decision task, colour Stroop task, Simon task, 6 subsets from the Test of Everyday Attention (map search, elevator task with distraction, elevator task with reversal, visual elevator task, telephone search task, dual telephone search task), and trail making task. Accuracy and reaction time (RT) data from each task was combined to create a cognitive profile, consisting of a total of 19 measures, for each participant.

Missing values were imputed using a *best guess* estimate after having been grouped by age group, language group, sex, and years in education. Any remaining missing values after the best guess estimation (i.e., the person may have ended up with a group combination on their own, thus limiting the ability to estimate realistic data), were imputed using a simple unitary imputation method (grand mean).

### Criterion

As there is no established or consistent definition of bilingualism (Dick et al., 2019), two measures were considered when developing the criterion. The first definition measured was a categorical self-identification “Are you bilingual?”; this evenly split the data (monolinguals *N* = 109, bilinguals *N* = 106). This is consistent with the methodology in the literature (Dick et al., 2019), however it is a rather liberal definition as it fails to provide information regarding the participants’ relationship with their language. To mitigate the risk of oversimplification and to take a more detailed assessment of bilingualism, 3 continuous indicators of bilingualism (frequency of use, exposure, and subjective proficiency) were grouped using a k-means clustering algorithm. The method reduces the three variables to one categorical variable with k groups, maximising between-group variance and minimising within-group variance. It was optimised with respect to the Silhouette value, and predicted 2 clusters that mapped closely, but not perfectly, onto the bilingual factor, with 126 being classified as “monolinguals” and 89 classified as “bilinguals”. Frequency of use was measured using a Likert scale with 7 levels ranging from 0 (“hardly ever or never”), to 6 (“every day multiple times a day”).

Language Exposure was measured by providing the participant with 7 examples of activities and asking them to indicate in which language they mostly engage with that activity - Welsh, English, or both. Given the ubiquity of English, those who answered ‘Welsh’ or ‘both’ were given a score of 1, and those to answered ‘English’ a score of 0 (Dick et al., 2019). Their exposure score was calculated as a sum of their answers.

In addition, participants self-rated their proficiency in both Welsh and English in Writing, Reading, Speaking, and Listening. Ratings were out of 5 (0 = “no ability” – 5 = “fluent”), with the final score being a mean of the four domains for each language. With little variance within the English subjective proficiency rating, only the Welsh subjective proficiency rating was included in the cluster analysis. Two non-language criterion were also modelled as a means to measure the validity of the machine learning algorithm and to act as a benchmark for the bilingualism models. The criterion were a) age, split by young (18-59) and older (60-90), and b) education, split by those who have achieved a higher education degree and those who have not.

### Cognitive Profile – Predictors

The cognitive profile was represented by 19 dependent variables derived from the 10 tasks the participants completed. As between differences can be more salient depending on the measure used, a combination of reaction times and accuracy scores were included.

#### Lexical Decision

Bilinguals have been found to be weaker in lexical access tasks, demonstrating reduced vocabularies in either one of their languages or increased reaction times in naming. The lexical decision task employed was replicated from Meyer and Schvaneveldt (1971), and involves participants being presented with a single word or pseudoword on a screen at a time. Participants were required to indicate, through specific key response, whether they believed the string was a real word or a pseudoword. 60 stimuli (40 real words and 20 pseudowords) appeared on screen until the participant made their response, with each one followed by a fixation cross for 2000ms. No feedback was given to the participants between trials. Only responses between 200ms and 5000ms were considered for the analysis. Participant performance was measured in two ways (i) mean reaction time (RT) for correct responses in ms, and (ii) d’ prime score (hit rate – false alarm rate).

#### Simon Task

Bilinguals have been shown to have an advantage in attention and inhibition tasks as measured by the Simon task (Simon and Rudell, 1967). It is claimed that the constant maintenance of two language systems simultaneously, results in increased activation of executive function processes, enhancing the individual’s ability to utilise domain general cognitive resources in non-language driven tasks. Simon tasks loads on executive function in a way that is sensitive to bilingual advantages. The traditional version of the Simon task was used in this experiment. Participants were presented with either a blue or red circle on either the left or right side of a computer display. They were instructed to press a corresponding key every time they saw a circle of a given colour, regardless of which side of the screen it was presented on (e.g., “A” key for blue and “L” for red). The keys were deliberately chosen as to be either congruent or incongruent to the placement of the coloured circles onscreen. In total, 80 congruent trials were displayed (both the presented circle and corresponding response key were on the same side), as well as 80 incongruent trials (the circle was presented on the opposite side of the screen to the corresponding response key). Participants were measured on their (i) mean accuracy for congruent and incongruent trials (2 separate scores out of 80), (ii) mean RT for correct responses for congruent and incongruent trials (2 separate scores measured in ms), and (iii) the Simon Effect for accuracy and RT, which were calculated as the mean accuracy from the incongruent trials subtracted from the mean accuracy of the congruent trials, and the mean RT for congruent trials subtracted from the mean RT for incongruent trials, respectively. This resulted in a total of 6 predictors.

#### Stroop Task

Like the Simon task, the Stroop task (Stroop, 1935) is also reported to load on response inhibition and effectively demonstrate the bilingual advantage. The colour Stroop task consisted of names of 8 primary colours being presented on a screen, written in congruent or incongruent font colour. 56 trials were presented in random order with equal number of congruent and incongruent trials. For the congruent trials the word matched its font colour (“RED” written in red), while incongruent trials displayed words in different font colour (“RED” written in blue). Participants were required to name the colour the word was written in and ignore the colour name on the screen. Input was received through a microphone which would record reaction time and move participants onto the next trial. The stimuli remained on screen until the participant responded, with a fixation cross between trials that was presented for 2000ms. Any recorded responses that weren’t between 200ms and 5000ms were excluded from the analysis. Participants were assessed on the mean Stroop RT for incongruent trials and the Stroop effect in ms (RT congruent trials – RT incongruent trials).

#### Test of Everyday Attention

The Test of Everyday Attention is a battery of cognitive tasks by Robertson et al. (1994), of which 6 of the 8 subsets were presented to participants. They assess five cognitive areas: (i) 2 measures of visual search (Subset 1, map search; Subset 6, telephone search) which measures the number of targets, out of a total of 80, circled on a map in 2 min; and the amount of time taken to circle a number of symbol pairs in a mock phone book divided by the number of correctly circled pairs, out of 20 (time-per-target), respectively; (ii) selective attention (Subset 3, elevator counting with distraction) as measured by the number of accurately counted sequences of auditory tones, out of 10; (iii) visual attentional switching (Subset 4, visual elevator) as measured by a the amount of time taken to correctly complete a puzzle in which the participants would need to mentally count a sequence elevator door pictograms, count backwards when they came across a pictogram of an arrow pointed up, and switch if they reached an arrow in the reverse direction, resulting in average time-per-switch score; (iv) auditory attentional switching (Subset 5, elevator counting with reversal) as measured by an auditory series similar to subset 3, but with an additional 2 tones requiring the participant to count-backwards or forwards depending on the pitch, scored as accuracy counted sequences out of 10; and (v) divided attention (Subset 7, telephone search dual task) as measured by the dual-task detriment score. The dual-task detriment score was calculated as the difference between the time taken to complete the telephone search task in Subset 6, and the time taken to complete a second telephone search task while also needing to accurately attend to an auditory counting task; finally, the score was modified with respect to accuracy in both the visual and auditory tasks. It has been noted that as tasks become more cognitively demanding, language group differences become more pronounced, as bilinguals are able to more efficiently recruit additional resources from non-domain specific regions. This bilingual advantage was hypothesised to result in the tasks with greatest cognitive load (e.g., Subset 5, elevator counting with reversal; and Subset 7, telephone search dual task), also being those with the highest predictive quality of bilingualism, especially in older adults (Bialystok et al., 2004).

#### Trails Making Test

The Trails Making Test (Partington and Leiter, 1949) is broadly characterised as a proxy for working memory or general processing speed, which are not commonly associated with bilingual advantage or disadvantage (Yang et al., 2018). However, evidence does exist that bilinguals outperform monolinguals on this task (Bialystok, 2010). This result was interpreted to be evidence of the broad non-domain specific effect of bilingualism. It is split into two main parts where participants have to sequentially connect 25 numbers (Trails A), and then alternate between numbers and letters in ascending order (Trails B). Participants are instructed to finish the trail as quickly as possible without crossing over any lines or lifting the pen off the page. The 2 predictor measures were included in the cognitive profile i) time to complete trails B in seconds, and ii) trails B-A in seconds (time to complete trails B in seconds - time to complete trails A in seconds). Time to complete trails A was not included as it showed little between-subjects variation.

### Random Forest Classifier

To measure the influence of bilingualism on multiple tasks simultaneously, a binary categorisation machine learning algorithm was used. The algorithm uses the cognitive task performance (predictors) to predict a dichotomous variable (criterion) for each participant. Should the algorithm recognise a consistent pattern of cognitive performance, and accurately distinguish between two groups (e.g., monolingual and bilingual), it also generated a feature importance list that indicates the importance of each predictor in making its decision. This helped to determine if there is a domain specific or a more domain general effect.

The algorithm chosen was the random forest classifier (Breiman, 2001), as it has been shown to have a high levels of accuracy and sensitivity in comparison to other classifiers (Maroco et al., 2011; Austin et al., 2013), and can be optimised relatively easily. The random forest model makes its classification through first using random bootstrapped sample of predictors from the training data to grow a decision tree. The decision tree uses arguments (e.g., Stroop reaction time < 200 ms) to split the data in what it believes to an effective way of dividing the levels of the criterion (bilinguals from monolinguals). The algorithm will then repeat the process by continuing to include predictors, and dividing the data, until such a point that only members of one group (bilinguals or monolinguals) are together in a leaf. However, a single decision tree is an inaccurate tool for predictive learning as it is unable to make predictions beyond the data on which it was created. Therefore, the random forest algorithm grows multiple unique trees using this method, selecting random sub-samples of predictors for each tree. When testing for accuracy, an unseen sub-sample of participant responses is passed through all the decision trees, and a majority vote classifies them as either one group or the other (bilingual or monolingual).

The classifier was trained and validated on 80% of the data and then tested on the remaining 20% of the total dataset. The data were analysed using the Scikit−Learn33ML library (Pedregosa et al., 2011), written in Python programming language, and JASP statistical analysis program (JASP Team, 2019). Models were optimised with respect to Out-of-bag accuracy, by allowing number of trees and predictors per split to vary. The models’ performance were assessed using the following: overall classification accuracy, precision, recall, F1, and area under curve (AUC). Accuracy and AUC provide straightforward assessments of how well a classification algorithm performed by measuring the proportion of correctly identified targets. However, there are examples where accuracy can present an over optimistic view of the algorithm’s performance. In cases of imbalanced groups, accuracy and AUC can be misleading due to the algorithm prioritising the more abundant class. Precision and recall can help in providing a more complete picture. Precision represents the proportion of positive predictions that are correct, and approaches 1 as the number of false positives approaches 0. Recall represents the proportion of the relevant class (e.g., bilinguals) that was correctly predicted, and approaches 1 as the number of false negatives approaches 0. F1 considers both precision and recall in a single statistic, and only approaches 1 when both precision and recall also approach 1. By accounting for false positives and false negatives the F1 statistic can provide crucial information regarding whether the accuracy scores are a valid measure of performance, particularly in imbalanced groups.

Finally, as it has been argued that bilingual advantage may not be as salient within younger cohorts (Bialystok et al., 2016), modelling was repeated using only the older participants (60-90 years old).

## Results

18 predictor variables were used to classify 4 criterion variables. A full list of the predictors used, and their descriptive statistics can be found in Table 1.

**TABLE 1:** Means and standard deviations for demographic, language and cognitive measures split by bilingualism.

	Bilingual	Monolingual
		
	Mean (SD)	Mean (SD)
**Language and Demographic measures**		
Age (years)	47.62 (20.83)	49.03 (23.42)
MOCA (score)	27.79 (1.97)	27.91 (1.84)
Education (years)	15.47 (3.09)	15.54 (3.18)
Frequency of use (/6)	4.63 (2.1)	0.36 (0.94)
Language Exposure (/7)	4.44 (2.13)	0.19 (0.63)
Self-rated proficiency (Welsh/5)	4.25 (1.01)	0.47 (0.64)
Self-rated proficiency (English, /5)	4.72 (0.58)	4.68 (0.7)
**Predictors**		
English lexical decision RT (ms)	1211.7 (434.46)	1112.85 (402.18)
English lexical decision accuracy (d prime)	3.05 (0.82)	2.98 (0.79)
Simon task congruent accuracy (/80)	71.56 (16.24)	71.13 (15.95)
Simon task congruent RT (ms)	559.62 (109.27)	570.3 (127.12)
Simon task incongruent accuracy (/80)	69.28 (17.34)	68.69 (17.02)
Simon task incongruent RT (ms)	576.02 (109.02)	592.29 (131.47)
Simon effect accuracy	2.27 (6.96)	2.44 (10.9)
Simon effect RT (ms)	16.4 (40.98)	22 (60.72)
Trails B (seconds)	61.74 (31.17)	66.59 (34.93)
Trails B-A (seconds)	32.37 (25.42)	35.34 (29.57)
Sub 1: Map Search (/80)	64.09 (12.35)	66.41 (10.88)
Sub 3: Elevator counting with distraction accuracy (/10)	8.08 (2.53)	7.96 (2.62)
Sub 4: Visual elevator timing score (ms per switch)	3818.99 (1800.98)	3885.12 (1937.48)
Sub 5: Auditory elevator with reversal accuracy (/10)	4.94 (3.59)	5.12 (3.11)
Sub 6: Telephone Search (ms per target)	2877.96 (959.24)	2851.5 (862.67)
Sub 7: Telephone search dual task, dual task decrement	2303.6 (3244.89)	1946.2 (2376.86)
Stroop task English incongruent RT (ms)	874.25 (175.79)	889.58 (195.86)
Stroop effect, English (ms)	53.41 (141.28)	37.99 (144.16)

*”Sub #” refers to the subsection within the Test of Everyday Attention.*

### Language Group Differences and Bilingualism Indicators

Of the monolinguals, as expected, some participants reported exposure to other languages in their subjective report; however, this was never above basic comprehension. The two groups were significantly different across all bilingual indicators. In frequency of use (as measured through a Likert scale ranging from 0 to 6), monolinguals (Mdn = 0, IQR < 0.001) scored significantly lower than bilinguals (Mdn = 6, IQR = 2) *U* = 916.5, *p* < 0.001, Rank-Biserial Correlation −0.84. To put this in perspective this ranks the average bilingual in the sample as speaking their languages “multiple times a day” with some variation, while the monolinguals reported using Welsh “hardly ever/never” with next to no variation.

For Language Exposure, there was a similar pattern with a highly significant difference between monolinguals (Mdn = 0, IQR < 0.001) and bilinguals (Mdn = 5, IQR = 3), *U* = 493.5, *p* > 0.001, Rank-Biserial Correlation −0.91. Here participants were scored on the sum number of activities where they engaged primarily in English, Welsh or Both. Lower scores represented predominantly English speakers, higher scores represent greater experience with Welsh or both languages equally. Potential scores ranged from 0 (No exposure to Welsh) to 7 (Highly immersed in either Welsh or both languages equally). As stated before, Welsh and both languages were grouped together due to the ubiquity of English in Wales with only 20% of the country speaking the language, even in isolated communities that predominantly use Welsh it would be impossible to navigate work and social environments without daily English exposure.

When participants rated their own proficiency in writing, reading, speaking and listening, there was no significant language group difference between the mean self-rated proficiency scores for English, with all participants scoring near ceiling (Bilingual: *M* = 4.72, *SD* = 0.58; Monolingual: *M* = 4.68, *SD* = 0.70); t(208.59) = 0.41, *p* = 0.68, Cohen’s *d* = 0.06. In contrast, the Welsh language proficiency scores showed a highly significant difference between the two groups (Bilingual: *M* = 4.25, Monolingual: *M* = 0.47); *t*(176.76) = 32.68, *p* < 0.001(*p* = 7.85_*e*_-77, Cohen’s *d* = 4.47).

There were no significant differences between bilinguals and monolinguals on age (years) (Monolinguals: *M* = *49.03, SD* = 23.42, Bilingual: *M* = 47.62, *SD* = 20.83), Welch’s *t*(211.32) = 0.46, *p* = 0.64, Cohen’s *d* = 0.063. Nor was there was a significant difference in years in education between monolinguals (*M* = 15.54, *SD* = 3.18) and bilinguals (*M* = 15.47, *SD* = 3.09), Welch’s t(213) = 0.16, *p* = 0.87, Cohen’s *d* = 0.022.

### Language Cluster Analysis

To incorporate a more robust and nuanced definition of bilingualism as a predictor, a k-means cluster analysis was applied to bilingualism indicators (frequency of use, exposure and subjective proficiency scores for Welsh) (von Bastian et al., 2016). K-means clustering produces a categorical variable with k groups with minimal within-group variance and maximum between-group variance. As the k-means algorithm is sensitive to scaling, the three variables were standardised using z-scores. 500 iterations were conducted with the number of clusters allowed to vary between 1 and 10, and was optimised with respect to the silhouette score. The three indicators of bilingualism were reduced to one dichotomous variable, the algorithm favouring a 2 cluster solution. Cluster 1 (*N* = 125) included primarily monolingual participants with some self-described bilinguals who reported lower frequency and proficiency in Welsh. Cluster 2 (*N* = 90) contained no monolinguals. This variable was labelled as “k-clustered bilingualism,” with cluster 1 and 2 being interpreted as monolingual and bilingual respectively.

No significant difference was observed between the clusters with respect to age Welch’s *t*(203.34) = 0.05, *p* = 0.96, Cohen’s *d* < 0.01, or years in education Student’s *t*(213) = −0.77, *p* = 0.44, Cohen’s *d* = −0.11.

### Single Main Effects

The individual effects of age, education, and bilingualism were first measured on the cognitive factors. Bilingualism was defined as two independent variables: Bilingualism (those who defined themselves as bilinguals), and k-clustered bilinguals (those identified as bilinguals using the k-means clustering method). All independent variables were dichotomous: (i) age (younger than 60 vs 60 and older), (ii) education (those who have achieved a higher education degree vs those who have not), and (iii) bilingualism (bilingual vs monolingual). Table 2 shows the results of conducted t-tests. Welch’s *t*-tests were used for comparisons where there was a violation of the equal variances assumption, otherwise a Student’s t-tests were used.

**TABLE 2:** Independent samples *t*-tests with Age, Education, language groups as depending groups.

	Age				Education				Bilingualism				k-cluster bilingualism			
				
Task	Test	*t* (df)	p	Cohen’s d	Test	*t* (df)	p	Cohen’s d	Test	*t* (df)	*p*	Cohen’s d	test	*t* (df)	*p*	Cohen’s d
English lexical decision RT (ms)	**Student**	**−3.41 (213)**	**7.76e −4**	**−0.47**	**Student**	**2.96 (213)**	**3.40e −3**	**0.41**	Student	−1.73 (213)	0.08	−0.24	Student	−1.88 (213)	0.06	−0.26
English lexical decision accuracy (d prime)	**Welch**	**−5.93 (210.84)**	**1.24e −8**	**−0.8**	**Student**	**−3.75 (213)**	**2.32e −4**	**−0.52**	Student	−0.59 (213)	0.56	−0.08	Student	−1.33 (213)	0.19	−0.18
Simon task congruent accuracy (/80)	Welch	1.68 (140.47)	0.1	0.24	Student	−0.1 (213)	0.92	−0.01	Student	−0.2 (213)	0.85	−0.03	Student	−0.23 (213)	0.82	−0.03
Simon task congruent RT (ms)	**Student**	**−9.96 (213)**	**1.97e −19**	**−1.37**	**Student**	**2.08 (213)**	**0.04**	**0.29**	Student	0.66 (213)	0.51	0.09	Student	0.3 (213)	0.76	0.04
Simon task incongruent accuracy (/80)	Welch	0.78 (159.81)	0.44	0.11	Student	−0.08 (213)	0.94	−0.01	Student	−0.25 (213)	0.8	−0.03	Student	−0.38 (213)	0.7	−0.05
Simon task incongruent RT (ms)	**Welch**	**−9.45 (163.17)**	**3.48e −17**	**−1.32**	**Student**	**2.11 (213)**	**0.04**	**0.29**	Student	0.99 (213)	0.33	0.13	Student	0.65 (213)	0.52	0.09
Simon effect accuracy	Student	1.61 (213)	0.11	0.22	Student	−0.03 (213)	0.97	−4.54e −3	Student	0.13 (213)	0.89	0.02	Student	0.32 (213)	0.75	0.04
Simon effect RT (ms)	Student	−0.21 (213)	0.83	−0.03	Student	0.16 (213)	0.87	0.02	Student	0.79 (213)	0.43	0.11	Student	0.81 (213)	0.42	0.11
Trails B (seconds)	**Welch**	**−6.42 (154.35)**	**1.58e −9**	**−0.9**	**Welch**	**3.17 (142.3)**	**1.89e −3**	**0.45**	Student	1.07 (213)	0.28	0.15	Student	1.07 (213)	0.29	0.15
Trails B-A (seconds)	**Welch**	**−4.82 (148.91)**	**3.48e −6**	**−0.68**	**Welch**	**3.07 (135.82)**	**2.56e −3**	**0.44**	Student	0.79 (213)	0.43	0.11	Student	0.74 (213)	0.46	0.1
Sub 1: Map Search (/80)	**Welch**	**8.2 (181.45)**	**4.35e −14**	**1.14**	Student	−1.2 (213)	0.23	−0.17	Student	1.46 (213)	0.15	0.2	Welch	1.51 (165.25)	0.13	0.21
Sub 3: Elevator counting with distraction accuracy (/10)	**Welch**	**2.47 (170.44)**	**0.01**	**0.35**	Student	−1.52 (213)	0.13	−0.21	Student	−0.35 (213)	0.73	−0.05	Student	−0.8 (213)	0.42	−0.11
Sub 4: Visual elevator timing score (ms per switch)	**Welch**	**−4.07 (151.65)**	**7.45e −5**	**−0.57**	Welch	1.18 (115.88)	0.24	0.17	Student	0.26 (213)	0.8	0.04	Student	0.63 (213)	0.53	0.09
Sub 5: Auditory elevator with reversal accuracy (/10)	**Student**	**9.41 (213)**	**8.22e −18**	**1.29**	**Welch**	**−2.79 (205.19)**	**5.80e −3**	**−0.38**	Welch	0.38 (207.18)	0.7	0.05	Welch	−0.36 (166.71)	0.72	−0.05
Sub 6: Telephone Search (ms per target)	**Welch**	**−11.03 (159.93)**	**2.09e −21**	**−1.55**	**Welch**	**2.46 (157.88)**	**0.02**	**0.35**	Student	−0.21 (213)	0.83	−0.03	Student	−0.6 (213)	0.55	−0.08
Sub 7: Telephone search dual task, dual task decrement	**Welch**	**−3.35 (181.97)**	**9.73e −4**	**−0.47**	**Welch**	**2.42 (139.51)**	**0.02**	**0.35**	Student	−0.92 (213)	0.36	−0.13	Student	−0.35 (213)	0.73	−0.05
Stroop task English incongruent RT (ms)	**Student**	**−5.1 (213)**	**7.50e −7**	**−0.7**	Student	0.94 (213)	0.35	0.13	Student	0.6 (213)	0.55	0.08	Student	0.96 (213)	0.34	0.13
Stroop effect, English (ms)	**Student**	**−2.14 (213)**	**0.03**	**−0.29**	**Student**	**2.11 (213)**	**0.04**	**0.29**	Student	−0.79 (213)	0.43	−0.11	Student	−0.16 (213)	0.87	−0.02

*Bold represents significance (*p* < 0.05); Negative effect sizes represent mean scores where younger adults < older adults (Age), No higher education < higher education (Education), Monolingual < Bilinguals (Bilingualism and k-cluster bilingualism)*

Age groups were found to have the most consistent significant differences with younger adults outperforming older adults across all significant measures, apart from in Lexical Decision accuracy (d-prime) where older adults outperformed younger adults. Only four variables failed to reach significance (Simon task congruent accuracy, Simon task incongruent accuracy, and Simon effect for accuracy and reaction times (RT); all *p*’s > 0.1). Education showed mixed results with 10 of the 18 variables demonstrating significant difference between those with a higher education and those without (Lexical Decision RT and accuracy, Simon task congruent and incongruent RT, Trails-B RT, Trails B-A, Test of Everyday Attention (TEA) Subset 5, TEA subset 6,TEA subset 7, and Stroop effect; all *p*’s < 0.05).

Neither of the bilingualism variables demonstrated significant effects on any of the cognitive measures. Taken on its own this highlights the difficulty of demonstrating bilingualism’s direct influence on cognitive performance. However, this method has its limitations, such a large number of comparison tests and with no corrections increases the chances of statistical error. Additionally, as stated in the introduction the effects of bilingualism are fairly small, and difficult to measure as a single factor, therefore necessitating an analysis of the aggregate before drawing conclusions.

### Random Forest

The following analysis investigates whether the task-specific significant and non-significant main effects observed by the t-tests cumulatively translate to a generalised cognitive profile that can be discriminated into two discrete levels for each classifier. Models were built having been trained on 80% of the cognitive data, which then attempted to correctly classify the remaining 20% as one of the two dichotomous classes (e.g., bilingual or monolingual). Each model was evaluated primarily based on overall classification accuracy, Area under the curve and F1 score. Feature importance tables were also presented for successful models to give insight into which cognitive predictors influenced the algorithm’s decision, and if it was domain focused, or more general.

#### Predicting Age

As the factor resulting in the largest and most consistent significant main effects, according to the independent t-tests, age was modelled first. This was in part to determine the effectiveness of applying random forest classification as method for analysis to this type of problem. Given the relatively large between-group differences for each of the cognitive measures, had inaccurate models been generated, it would suggest that random forest was inappropriate for this type of study. Alternatively, with successful models, the accuracy could operate as a benchmark by which to compare the predictive models for bilingualism and education.

**FIGURE 1:**
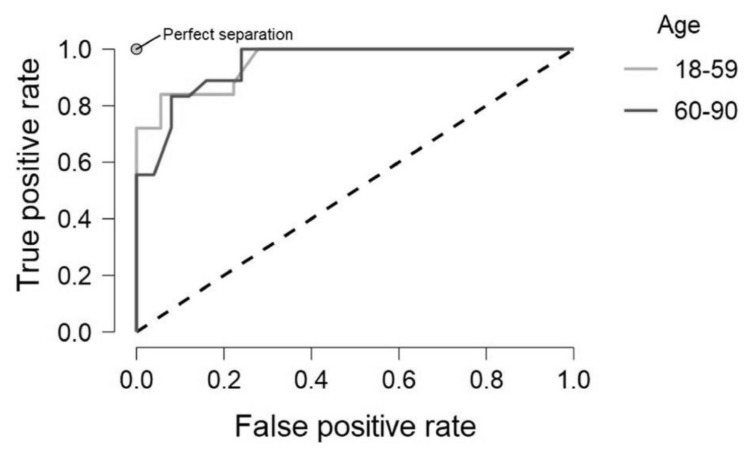
ROC plot demonstrating the true and false positive rate for both age groups.

The random forest classifier showed high levels of accuracy correctly predicting 93% of test cases. Additionally, the high F1 score indicates that the algorithm didn’t prioritise one class at the expense of the other (Table 3). Figure 1 visually demonstrates the relationship between True positive rate (correctly identified members of the target class) and False positive rate (incorrectly identified members of the non-target class). Plot lines for more successful models tend towards the top left, high true positive rate and low false positive rate. This represents a model that is both accurate for the target class and non-target class. The dashed line represents a 1:1 ratio between true positive and false positive, suggesting a model that prioritised one class at the expense of the other. This is usually the result of unbalanced indistinct classes, where the most accurate strategy was to label all instances as members of the same class. Anything lower than the dashed line represents a model that developed a poor strategy resulting in a greater rate of false positives than true positives.

**TABLE 3:** Evaluation Metrics for random forest classifier predicting age.

Model Name	Test Accuracy	Precision	Recall	F1 Score	AUC
Model Age	0.93	0.94	0.93	0.93	0.97

*Age was separated dichotomously into younger (18-59) and older (60-90) adults. Area Under Curve (AUC) is calculated for every class against all other classes.*

Finally, Table 4 shows the generated feature importance list for this model. It can be observed that the most important predictors in determining which age group each cognitive profile belonged to, were also the variables with the largest effect size. As both reaction time and accuracy data was used to create the models, effect sizes were either positive or negative. Younger adults had quicker reaction times, and higher accuracy scores. Direction of the effect (whether younger adults out preformed older adults, or vice versa) played no role. The mean decrease in accuracy represents by how much the model’s classification accuracy would be expected to lower with the removal of a given predictor. Higher mean decrease in accuracy indicates greater relative importance.

**TABLE 4:** Subset of the feature importance table for random forest classifier of age and corresponding effect sizes.

Task	Mean decrease in accuracy	Effect size (Cohen’s *d*)
Sub 6: Telephone Search (ms per target)	0.08	−1.55
Sub 1: Map Search (/80)	0.04	1.14
Simon task incongruent RT (ms)	0.02	−1.32
Sub 5: Auditory elevator with reversal accuracy (/10)	0.02	1.29
Simon task congruent RT (ms)	0.01	−1.37

**Note*. Top five features were included in this subset. Negative effects indicate that younger adults were lower than older adults. All effects were significant using a *t*-test.*

#### Predicting Bilingualism

Two models were initially conducted for bilingualism. Model 1 had self-identified bilinguals as the target variable, while Model 2 had the cluster identified bilinguals as the target variable. Coincidentally both models achieved an overall test accuracy of 0.47, or just under chance, indicating that neither were able to reliably discriminate between bilinguals and monolinguals. Further evaluation metrics, presented in Table 5, support this with low F1 and AUC scores.

**TABLE 5:** Evaluation Metrics for random forest classifier models predicting bilingualism.

	Test Accuracy	Precision	Recall	F1 Score	AUC
Model 1	0.47	0.51	0.47	0.47	0.54
Model 2	0.47	0.56	0.47	0.49	0.45
Model 1 (older)	0.44	0.7	0.58	0.57	0.64
Model 2 (older)	0.5	0.77	0.47	0.54	0.61

**Note*. Model 1 = self-identified bilingualism, Model 2 = k-cluster defined bilingualism. “older” signifies that the model contained from only older participants (60-90 years of age).*

**FIGURE 2:**
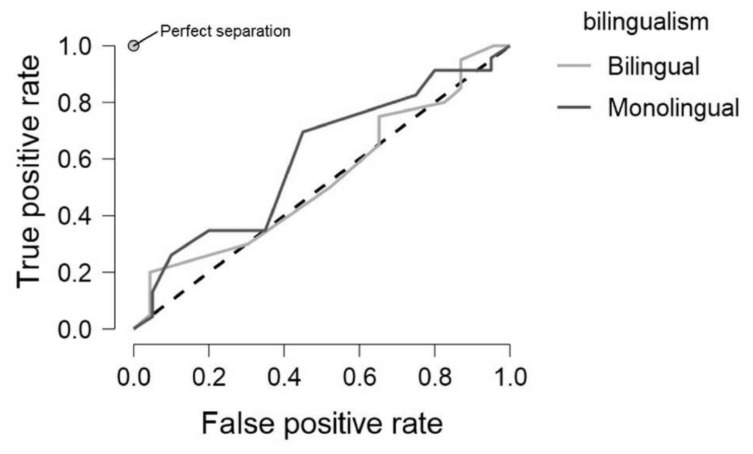
ROC plot demonstrating the true and false positive rate for self-identified bilinguals and monolinguals for Model1 containing both younger and older participants who self-identified as bilinguals.

To determine whether bilingualism is more a feature of cognitive reserve, providing a compensatory advantage to older adults that would not be observed in the younger cohort, older adults (60–90 years old, *N* = 97), were tested independently. However similar results were observed, with Model 1 (self-identified bilingualism) and Model 2 (cluster defined bilingualism) demonstrating test accuracy of 0.44 and 0.50, respectively with similar performance metrics (Table 5). Precision is the ratio of correctly classified members of a class to the total of predicted members of that class, for example the ratio between number of bilinguals correctly predicted to be bilingual vs the total number of predicted bilinguals. This was higher for the older sample in comparison to the younger sample. However, with low recall and F1 scores this may indicate that the models favoured one class at the expense of the other. Figure 2. Demonstrates the true positive rate against the false positive rate for identifying bilinguals in model 1. There is a stark difference between the predictive power of age (Figure 1), and bilingualism, with the ratio displayed here climbing at a near 1:1 ratio.

#### Higher Education

**FIGURE 3:**
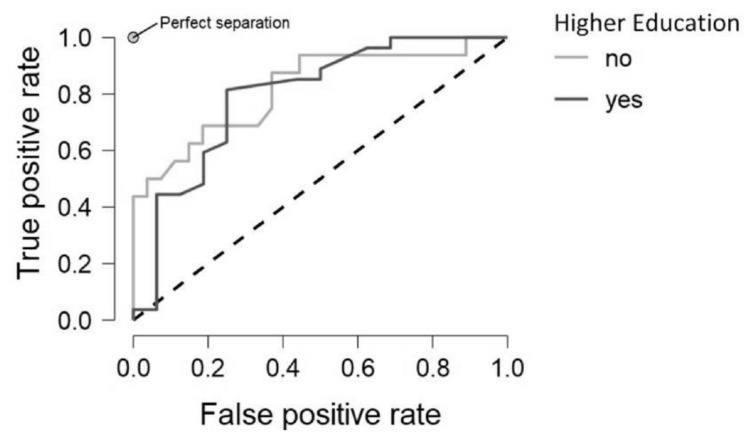
ROC Curves Plot for Model Education, demonstrating the true and false positive rate for those with and without a higher education, the model contains data from both younger and older participants.

Lastly the algorithms were modified to identify those with a higher education degree. As education has been reported to contribute to cognitive ability generally, as well as to cognitive reserve, it was predicted that the random forest algorithm would be able to accurately classify those with a higher education degree, particularly with older adults. This was found to be the case with an overall test accuracy of 77% for the entire cohort, which increased to 83% when only considering the older adults. Importantly, the reasonably high F1 score (Table 6) indicates that the algorithm was able to manage the imbalanced groups and maintain a high-test accuracy without overly prioritising the majority class (those with a higher education qualification). This demonstrates that, even with mixed significance across the tasks, the random forest algorithm was able to determine which participants had a higher education. Figure 3 shows the ROC plot for the model based on the entire cohort. Here it can be seen that education was not as powerful a predictor as age (Figure 3), however it is far more accurate in comparison to bilingualism.

**TABLE 6:** Evaluation Metrics for random forest models with higher education as target.

Model	Test Accuracy	Precision	Recall	F1 Score	AUC
Model Education	0.77	0.76	0.77	0.76	0.84
Model Education (older)	0.83	0.88	0.83	0.83	0.91

*Model Education contained data from younger and older participants, Model Education (older) contained data from just the older participants (60-90). Area Under Curve (AUC) is calculated for every class against all other classes.*

As with age, those variables that demonstrated the largest effect size also were the greatest contributors to the random forest decision making when classifying those with higher education (Table 7). Interestingly it was the more cognitively demanding task as well as the task reliant on knowledge of vocabulary that were of greatest importance.

**TABLE 7:** Subset of the feature importance table and corresponding effect sizes for random forest classifier of education.

Task	Mean decrease in accuracy	Cohen’s *d*
English lexical decision accuracy (d prime)	0.017	−0.52
Trails B (seconds)	0.014	0.45
Trails B-A (seconds)	0.011	0.44
English lexical decision RT (ms)	0.007	0.41
Sub 7: Telephone search dual task, dual task decrement	0.004	0.35

**Note*. Subsets contains top 5 most important features. Negative effects sizes indicate that RT/accuracy for those with no higher education were less than those with a higher education. All effects were significant using a *t*-test.*

## Discussion

The present study investigated bilingualism as pervasive cognitive stimulation that fundamentally alters brain performance and function (Kroll and Bialystok, 2013). Proponents of the bilingual effect argue that by maintaining two active languages bilinguals allocate cognitive resources in a manner that is quantifiably different to matched monolinguals (Bialystok, 2009). Crucially, these cognitive changes have been claimed to not be restricted to a single domain, instead building on a network of processes, and thus cannot be studied using a univariate approach. While this does an offer explanation for the large number of conflicting findings, it also makes any attempt to study the true impact of bilingualism very challenging using traditional compartmental experimental designs focusing on a single cognitive function (Bialystok, 2016). Therefore, to address this issue, a more inclusive ‘cognitive profiling’ approach was undertaken, observing participant cognitive performance across 10 different tasks in order to predict a dichotomous variable. The selected tasks had shown bilingual differences in prior research, and the 19 derived predictors were proxies for cognitive abilities that are argued to demonstrate bilingual advantages or disadvantages (Bialystok, 2009; Calvo et al., 2016). These cognitive abilities, as well as multiple indicators of bilingualism, were analysed simultaneously using machine-learning algorithms. As machine learning represented a novel ‘bottom up’ analytical approach in cognitive-behavioural research, additional cognition modulating classifiers, age and education, were also modelled as a form of cross-validation.

When presented with the participants’ cognitive profiles a random forest classifier demonstrated high accuracy and specificity for discriminating between younger and older adults. This was expected, as not only was cognitive decline in older adults supported by the current academic consensus (Ardila et al., 2000), but also by the task specific significance testing (which showed highly significant age related effects for all the tasks). Younger adults performed better on executive control tasks and were faster in all tasks (lower reaction times), although older adults were significantly better in lexical decision accuracy measures. Importantly it was the between-group effect sizes that were discovered to play an integral role in determining the importance of a predictor in the classification model. Intuitively, predictors with the largest between group effect size were also those that resulted in the highest mean decrease in accuracy. Highlighting the importance of reporting effect sizes when making predictive statements about significant findings. Taken together it was established that while the algorithm was not perfect, it was highly effective at classifying age group differences in our overall sample.

The same analytical approach found that level of education was also consistently accurately classified. Importantly, while cited in the literature as having a profound effect on cognitive performance, the task specific t-tests were more mixed than with age. Despite this, the algorithm was able to identify the variables with the greatest effects and use them to make accurate predictions at a rate greater than chance. This helps to demonstrate the validity of the machine learning algorithm approach in identifying populations with distinct cognitive profiles, even under conditions where not all the predictors show significant between group differences. It also supports the literature arguing that education results in an observable pattern of cognitive performance, particularly with respect to age related cognitive decline (Capitani et al., 1996; Ardila et al., 2000). This study supported the compensatory benefit of education in older adults as a function of cognitive reserve, as the algorithm was more accurate when only the older adults were modelled (Capitani et al., 1996; Stern, 2012).

Despite success for two key variables (age and education), we found no evidence supporting cognitive ability predicting bilingualism. The random forest algorithm was not shown to be effective at classifying monolingual and bilingual participants based on their cognitive profile, maintaining an accuracy rate of around 50%. Several models were conducted in order to account for some of the criticisms with bilingual research and address common confounds argued to obfuscate the bilingual effects (cf. (Kroll and Bialystok, 2013). Bilingualism was measured using 4 methods, multiple cognitive functions were examined simultaneously and homogenic cohorts of bilinguals and monolinguals were used (e.g., consistent languages spoken within groups, similar proficiency). Additionally, separate models were conducted including a broad age range, followed by a focus on older participants. However, despite these approaches we found no evidence indicating an effect of bilingualism as a significant predictor of cognitive differences among natural, balanced, high proficiency bilinguals (Zahodne et al., 2014). Instead, this study seems to echo the conclusions of more recent, highly powered, publications which have failed to find the bilingual advantage through more traditional designs and statistical inference (Paap and Greenberg, 2013; Paap et al., 2015b; von Bastian et al., 2016). Given these findings, this study concludes that the bilingual effects previously reported are not as broad or robust as once thought (Lehtonen et al., 2018), nor are the inconsistencies adequately explained by a domain general effect (von Bastian et al., 2016).

Instead, we argue that as no effect was found using a large cohort that was reasonably homogeneous both within and between groups in language and socio-demographic qualities, the bilingual effect may be context dependent (cf. Calvo et al., 2016). That is, that the bilingual effects found in the prior literature are the product of specific factors related to the bilingual experience, rather than purely a consequence of being bilingual. Certain factors were addressed here, including; age of acquisition, frequency of language use, level of relative exposure to each language, number of languages spoken, monolinguals’ experience with other languages, language proficiency, consistent language pairs, and age. However, this is not an exhaustive list (cf. Calvo et al., 2016). An alternative, and popular, explanation for the bilingual effect is immigrant status, where the cognitive stimulation involved in adapting to new culture, and potentially needing to learn a new language as part of that process, may account for the cognitive differences between bilinguals and monolinguals (Kousaie et al., 2014; Paap et al., 2015a; de Bruin, 2019). From this, other “hidden factors” are also proposed to potentially be conflated with bilingualism (Hilchey and Klein, 2011). Notably socio-economic status has been shown to influence cognition independent of language ability (Mezzacappa, 2004), and it is commonly found to be a significant factor differentiating immigrant and native populations (Rivera Mindt et al., 2008). While not directly controlled for here, both monolingual and bilingual participants were largely sampled from the same population with no reason to assume significant differences in socio-economic status between groups (Gathercole et al., 2014). Education, which has previously been used as a proxy for socio-economic status in bilingual research (Bialystok et al., 2004, 2008), was measured, and no bilingual/monolingual differences were found. As level of educational attainment was predicted with a much higher level of accuracy than bilingualism, it suggests that bilingual effects may, at least in part, be a product of associated variables.

Large scale meta-analyses also corroborate this assessment, concluding that while bilingualism is widely accepted to have functional benefits to cognitive processing, this is not borne out in the literature when publication bias is accounted for de Bruin et al. (2015). However, publication bias is not unique to bilingualism research, and the incorporation of papers that had failed to reach publication into the meta-analysis may also incorporate any methodological limitations with that work (Bak, 2016). Additionally, bilingual effects have been shown not to manifest as prominently in younger healthy adults, therefore the majority of research on this population would be expected to yield null results (Antoniou, 2019). This study attempted to mitigate this risk by modelling older adults, both as part of a broader age demographic and also separately. However, it should be noted the sample size used in this study is relatively small for machine learning studies in the fields of computer science and finance, which usually incorporate several thousand data points for both learning and testing. While the sample is relatively large compared to similar studies (von Bastian et al., 2016) and those that have found significant bilingual effects (Bialystok et al., 2008, see also Paap and Greenberg, 2013), and although a null effect for bilingualism was found, our bootstrapping techniques and hyperparameter optimisation resulted in successful models for predicting age, and education. Future research may seek to recruit larger sample sizes, but it appears unlikely at this time that insufficient data can explain the lack of language group differences, particularly when taking into account the multiple non-significant t-tests. Instead, as the feature importance of the successful models mapped closely to the effect sizes of the independent *t*-tests, it may be that the cognitive advantages/disadvantages for bilinguals were so small under these conditions as to make them indistinguishable from monolinguals. This would also remain true for the compensatory effects in older adults.

Adequately accounting for the myriad of confounding variables, and definitions of bilingualism, represents a major challenge to researchers moving forward. Even bilinguals who are very similar in terms of languages spoken, age of acquisition and proficiency may demonstrate different cognitive performance based on how they use their languages (Green and Abutalebi, 2013). Therefore, not all ‘bilinguals’ would be expected to demonstrate the same cognitive benefits (Antoniou, 2019). Additionally, the degree to which other variables are conflated with bilingualism or are conveying similar benefits is still unknown.

This challenge could potentially be addressed from the more wide-spread utilisation of machine learning. Machine learning algorithms are able to take advantage of the highly dimensional nature of cognitive decline, bilingualism and their co-variates, and present a more holistic, nuanced, perspective for the field. This method is beginning to be more widely adopted by similar fields as a more pragmatic and interpretable framework, when dealing with complex problems without a univariate solution (Paulus et al., 2016). In this study, although bilingualism was not accurately predicted, success was observed for the classification of age, and education groups. By refining this approach and including a broader range of predictor variables, more robust models may be developed, able to reliably identify cognitive patterns of bilingualism. Based on these predictions it will become possible to develop a detailed theoretical model that maps the relationship between bilingualism and cognitive domains. While, one unifying factor is unlikely to resolve the debate surrounding bilingualism, future studies should aim to identify the key variables, or combination of variables, that contribute to cognitive differences between monolinguals and bilinguals.

## Data Availability Statement

The raw data supporting the conclusions of this article will be made available by the authors, without undue reservation.

## Ethics Statement

The studies involving human participants were reviewed and approved by Swansea University Board of Ethics. The patients/participants provided their written informed consent to participate in this study.

## Author Contributions

SJ did the conceptualisation, design of methodology, coding of experimental tests and analysis, data collection, analysis, writing of original manuscript, writing of the revised manuscript, and visualisation of data through tables and figures. JD-T wrote, reviewed, and edited the manuscript. JT did the conceptualisation, supervised the design and execution of the study, provided access to testing materials, and reviewed and edited the manuscript. All authors contributed to the article and approved the submitted version.

## Conflict of Interest

The authors declare that the research was conducted in the absence of any commercial or financial relationships that could be construed as a potential conflict of interest.
